# Higher in status, (Even) better-than-average

**DOI:** 10.3389/fpsyg.2015.00496

**Published:** 2015-04-28

**Authors:** Michael E. W. Varnum

**Affiliations:** Psychology, Arizona State UniversityTempe, AZ, USA

**Keywords:** SES, social class, better-than-average-effect, illusory superiority, self-enhancement

## Abstract

In 5 studies (total *N* = 1357) conducted online using Amazon's MTurk the relationship between socioeconomic status (SES) and the better-than-average effect (BTAE) was tested. Across the studies subjective measures of SES were positively correlated with magnitude of BTAE. Effects of objective measures (income and education) were weaker and less consistent. Measures of childhood SES (both objective and subjective) were positively correlated with BTAE magnitude, though less strongly and less consistently than measures of current subjective SES. Meta-analysis revealed all measures of chronic SES (with the exception of education) were significantly correlated with BTAE. However, manipulations of SES in terms of subjective status (Study 2), power (Study 3), and dominance (Study 4) did not have strong effects on BTAE magnitude (d's ranging from −0.04 to −0.14). Taken together the results suggest that chronic, but not temporary, status may be linked with a stronger tendency to overestimate one's abilities and positive traits.

## Introduction

How good of a driver are you? How friendly are you? How intelligent? If you're like most people, you're unlikely to say that you are “average” in any of these domains. In fact most people rate themselves as above average in terms of a wide range of skills, abilities, and positive traits ranging from cognitive abilities, to driving, to popularity to name just a few (Svenson, 1981; Alicke, 1985; Kruger and Dunning, 1999; Zuckerman and Jost, 2001; Roese and Olson, 2007). This phenomenon is known as the better-than-average effect (BTAE; Brown, 1986; Kruger and Dunning, 1999). This phenomenon has been demonstrated repeatedly and in a variety of domains. Yet it may be that some people are more prone to this bias than others. Recent studies have shown that people who are higher in socioeconomic status (SES), also tend to be higher in narcissism and entitlement (Piff, 2014), have higher self-esteem (Kraus and Park, 2014), and are more self-focused than those who are lower in SES (Na et al., 2010; Grossmann and Varnum, 2011; Varnum et al., submitted). Given these differences, one might expect that those who are higher in SES might also be more likely to have an inflated view of their skills, abilities, and positive traits. The present series of studies tested whether SES was associated with strength of BTAE. Consistent with previous findings regarding SES, it was predicted that high SES would be associated with a stronger tendency to rate the self as better than average.

SES has both subjective and objective components (educational attainment, income). Although these components often have comparable effects on psychological tendencies (for a review see Kraus et al., 2011), this is not always the case (Kraus et al., 2013). Further, a number of studies have focused on childhood SES background, sometimes finding parallel effects as those observed for current SES (Grossmann and Varnum, 2011; Varnum et al., 2012), sometimes finding stronger effects for childhood vs. current SES (Griskevicius et al., 2011). Further, subjective components of SES can be manipulated (Kraus et al., 2010; Miyamoto and Wilken, 2010), often producing parallel results to those observed for chronic markers of SES (Kraus et al., 2010; Miyamoto and Wilken, 2010). Yet it may be that for some psychological tendencies, one's chronic level of SES is more important than how one feels about their status in a given moment or situation. For example, some effects may be canalized, whereas other may be more dependent on immediate context. Thus, the second aim of the present series of studies was to compare the effects of different types of SES (subjective vs. objective, current vs. childhood, and temporary vs. chronic) on BTAE.

Study 1 tested the association between various measures of SES and BTAE. Studies 2–4 manipulated various aspects of status and explored the effects of these manipulations of BTAE, as well as testing links between measures of chronic SES and BTAE. Study 5 was a replication of Study 1 that also tested self-construal as potential mechanism underlying the SES—BTAE link.

## Study 1

### Materials and methods

209 participants (67 f, 139 m, 3 did not provide gender or SES information) recruited through Amazon's MTurk completed the study online. Participants were paid $0.50 for taking part in the study. Only those who were US residents with a 95% or higher lifetime approval rating on MTurk were eligible to take part in the study. All studies were approved by the IRB at Arizona State University. Informed consent was obtained for all studies reported in this manuscript.

Participants were asked to rate themselves on 9 domains (memory, physical fitness, driving ability, honesty, sense of humor, attractiveness, intelligence, cooking ability) in terms of percentiles using slider bars anchored at the 1st percentile. These domains were selected to provide broad coverage of desirable traits, skills, and abilities in a number of different domains. The order in which items appeared on the page was randomized. Participants were given the following instructions:

“For each of the following skills, abilities, and attributes, please indicate where you stand in comparison to other Americans. For example if you think you indicate you are in the **50th percentile** that means you believe that **approximately 50% of Americans are higher than you** are on that particular dimension and **approximately 50% are lower than you** are on that particular dimension. The **lowest possible score** is the **1st percentile** (which means that you believe **99% of Americans are higher than you** on that particular dimension); the **highest possible score** is the **99th percentile** (with means that you believe **99% of Americans are lower than you** on that particular dimension).”

The responses to the 9-items (memory, physical fitness, driving ability, honesty, sense of humor, attractiveness, intelligence, friendliness, cooking ability) were averaged and the difference between that average and 50 was taken to create an index of BTAE (higher scores = rating oneself at higher than the 50th percentile). Reliabilities and means for each study are presented in Table 1.

**Table 1:** **Studies 1–5 Better than Average Effect (BTAE) by sample and condition**.

	**Condition Effect size of condition**	***N***	***M*(SD)**	**Cornbach's alpha (self-ratings on 9 dimensions)**	***t* (One sample test, mean self-ratings criterion = 50)**
Study 1		209	62.64 (11.56)	0.77	15.80^***^
Study 2	Total	318	63.20 (12.40)	0.77	18.98^***^
	Low Status	156	62.82 (12.58)		
	High Status	162	63.56 (12.26)		
	Cohen's *d* = 0.06				
Study 3	Total	305	63.49 (11.62)	0.75	20.28^***^
	Low Power	159	62.74 (11.45)		
	High Power	146	64.31 (11.79)		
	Cohen's *d* = 0.14				
Study 4	Total	316	64.82 (12.11)	0.77	21.45^***^
	Team Player	158	64.85 (12.41)		
	Leader	158	64.39 (11.85)		
	Cohen's *d* = −0.04				
Study 5		228	63.05 (12.62)	0.78	15.61^***^

Means represent average percentile self-ratings across 9 abilities and traits (ratings greater than 50 indicate the presence of BTAE).

*** p < 0.001.

Next, participants completed the MacArthur subjective social status ladder (SSS), on which they ranked their SES relative to other Americans on a 10-pt scale. Participants also provided data on their educational attainment on a 6-pt scale (“1” = “Did not complete high school,” “6” = “PhD, MD, JD”), indicated their income using a slider bar (range: 0–$500,000), and indicated which label best described their current SES and childhood SES on 5-pt scales (“1” = “Lower Class,” “5” = “Upper Class”), and their childhood family income (using a slider bar, range 0–$500,000). In addition participants answered demographic questions regarding gender, ethnicity, country of birth, and age.

### Results

Consistent with previous research, on the whole participants rated themselves as better than average (i.e., higher than the 50% percentile, see Table 1). Both subjective measures of current SES were correlated with stronger BTAE (SSS: *r* = 0.16, *p* < 0.05; Class (5-item measure): *r* = 0.22, *p* < 0.01), as were childhood subjective class (Childhood Class (5-item measure): *r* = 0.26, *p* < 0.001) and childhood family income (*r* = 0.16, *p* < 0.05). Neither educational attainment, nor current income were correlated with BTAE (*r*'s < 0.06, ns; see Table 2).

**Table 2:** **Correlations between various measures of social class and BTAE by sample**.

**Sample**	***N*^a^**	**SSS (ladder)**	**Class**	**Income**	**Education**	**Childhood class**	**Childhood income**
Study 1 Chronic SES	206	0.16^*^	0.22^**^	0.05	0.03	0.26^***^	0.16^*^
Study 2 SSS priming	318	0.16^**^^b^	0.13^*^	0.06	−0.01	0.07	0.08
Study 3 Power priming	305	0.25^***^	0.20^**^	0.11^x^	0.05	0.08	0.17^**^
Study 4 Leadership bogus feedback	314	0.33^***^	0.26^***^	0.12^*^	0.02	0.15^*^	0.17^**^
Study 5 Chronic SES (SCS also measured)	214	0.34^***^	0.17^*^	0.10	0.13^x^	0.22^**^	0.20^**^
Total^c^	1357	0.25	0.19	0.09	0.04	0.14	0.15

a Largest N for which there is data on an SES measure and BTAE; for Study 2 N's for correlations range from 316 to 318, for Study 3 N's for correlations range from 303 to 305, for Study 4 N's for correlations range from 311 to 314, for Study 5 N's for correlations range from 208 to 214.

b SSS measured as part of priming procedure.

c Correlations are mean R's weighted by sample size.

x p < 0.10,

* p < 0.05,

** p < 0.01,

*** p < 0.001.

## Study 2

### Materials and methods

318 participants (117 f, 196 m, 5 did not provide gender information) recruited through Amazon's MTurk completed the study online. An additional 17 participants completed the first part of the study (ranking themselves on the SSS ladder), but not subsequent portions of the study. Participants were paid $0.50 for taking part in the study. Only those who were US residents with a 95% or higher lifetime approval rating on MTurk were eligible to take part in the study.

The procedure was the same as in Study 1, with the exception that at the beginning of the study participants were asked to complete the SSS measure. The instructions varied such that in one condition (Low Status) participants were instructed to compare themselves with those at the top of the ladder, whereas in another condition they were instructed to compare themselves to those at the bottom of the ladder (High Status; Kraus et al., 2010). Participants were randomly assigned to experimental conditions, (Low Status *N* = 166, High Status *N* = 169).

### Results

Consistent with previous research, on the whole participants rated themselves as better than average (i.e., higher than the 50% percentile, see Table 1). As in Study 1, subjective measures of current SES were correlated with stronger BTAE (SSS: *r* = 0.16, *p* < 0.05; Class: *r* = 0.13, *p* < 0.05). No other measures of SES were correlated with BTAE (see Table 2). Participants in the two conditions did not differ in BTAE, *F*_(1, 316)_ = 0.28, ns. Those in the High Status condition ranked themselves higher on the SSS ladder (*M* = 4.75, *SD* = 1.64), than those in the Low Status condition (*M* = 4.57, *SD* = 1.70), however this difference failed to reach statistical significance, *F*_(1, 333)_ = 1.03, ns.

## Study 3

### Materials and methods

305 participants (126 f, 129 m) recruited through Amazon's MTurk completed the study online. An additional 3 participants completed the first stage of the study (the “episodic memory” portion) but not the second. Participants were paid $0.50 for taking part in the study. Only those who were US residents with a 95% or higher lifetime approval rating on MTurk were eligible to take part in the study.

The procedure was the same as in Study 1, with the exception that subjects were told they would complete 2 brief studies, the first on “episodic memory,” the second on “skills and abilities” (which followed the same procedure as Study 1). Using a procedure adapted from Miyamoto and Wilken (2010) Subjects were first asked to recall and briefly describe instance where they adjusted themselves to the wishes of another (Low Power), or where they influenced or changed another person (High Power). Participants were then asked to rate the extent to which they adjusted to others in the recalled situation and the extent to which they influenced others using a series of circles with varying degrees of overlap (Miyamoto and Wilken, 2010). Participants were randomly assigned to experimental conditions (Low Power *N* = 160, High Power *N* = 148).

### Results

Consistent with previous research, on the whole participants rated themselves as better than average (i.e., higher than the 50% percentile, see Table 1). As in Studies 1 and 2, subjective measures of current SES were correlated with stronger BTAE (SSS: *r* = 0.25, *p* < 0.001; Class: *r* = 0.20, *p* < 0.01). Childhood income was correlated with stronger BTAE (*r* = 0.17, *p* < 0.01), and current income was marginally correlated with stronger BTAE (*r* = 0.11, *p* < 0.1). No other measures of SES were correlated with BTAE (see Table 2). Participants in the two conditions did not differ in BTAE, *F*_(1, 303)_ = 1.39, ns. Participants in the High Power condition reported influencing others (*M* = 5.07, *SD* = 1.53), more than those in Low Power condition (*M* = 3.08, *SD* = 1.90), in the recalled interaction, *F*_(1, 306)_ = 100.97, *p* < 0.001. Those in the Low Power condition reported adjusting to others (*M* = 4.87, *SD* = 1.60), than those in the High Power condition (*M* = 3.64, *SD* = 1.78), in the recalled interaction, *F*_(1, 306)_ = 40.94.

## Study 4

### Materials and methods

316 participants (126 f, 185 m, 5 did not provide gender information, 2 did not provide any SES information) were recruited through Amazon's MTurk completed the study online. Participants were paid $0.50 for taking part in the study. Only those who were US residents with a 95% or higher lifetime approval rating on MTurk were eligible to take part in the study.

The procedure was the same as in Study 1, with the exception that subjects were told they would complete 2 brief studies, the first a “personality test,” the second on “skills and abilities” (which followed the same procedure as Study 1). In the first phase participants were told:

“In this study you will be asked a few brief questions about your personality. After you have answered these questions you will receive a personality profile based on your responses. These profiles are based on how responses to these items are linked to in depth assessments of people's traits and experiences. Previous research has suggested that people's patterns of answers on this very brief inventory can reveal a great deal about who they are.”

Participants then completed the 10-item Big 5 inventory (Gosling et al., 2003). Participants received bogus feedback (see Appendix), either describing their personalities as consistent with being a “leader” (*N* = 158), or a “team player” (*N* = 158). After receiving the feedback participants were asked to indicate the extent to which they felt the feedback was an accurate description of their personality on a 7-pt scale (“1” = “strongly disagree,” “7” = “strongly agree.”).

### Results

Consistent with previous research, on the whole participants rated themselves as better than average (i.e., higher than the 50% percentile, see Table 1). As in Studies 1–3, subjective measures of current SES were correlated with stronger BTAE (SSS: *r* = 0.33, *p* < 0.001; Class: *r* = 0.26, *p* < 0.001). Current income was correlated with stronger BTAE (*r* = 0.12, *p* < 0.05), as were childhood class (*r* = 0.15, *p* < 0.05) and childhood income (*r* = 0.17, *p* < 0.01). Education was not correlated with BTAE (*r* = 0.02, ns). Participants in the two conditions did not differ in BTAE, *F*_(1, 314)_ = 0.12, ns. A one sample *T*-test comparing participants' belief that the personality feedback they received was accurate (vs. the mid-point of the scale), showed that participants' did indeed believe the feedback that they received, *M* = 5.54, *SD* = 2.47, *t*_(315)_ = 11.06, *p* < 0.001.

## Study 5

### Materials and methods

228 participants (83 f, 125 m, 20 did not provide gender information, 14 did not provide any SES information) were recruited through Amazon's MTurk completed the study online. Participants were paid $0.50 for taking part in the study. Only those who were US residents with a 95% or higher lifetime approval rating on MTurk were eligible to take part in the study. The procedure was the same as in Study 1 with the addition of the 30-item version of the self-construal scale (Singelis, 1994).

### Results

Consistent with previous research, on the whole participants rated themselves as better than average (i.e., higher than the 50% percentile, see Table 1). As in Studies 1–4 subjective measures of current SES were correlated with stronger BTAE (SSS: *r* = 0.34, *p* < 0.001; Class: *r* = 0.17, *p* < 0.05). Education was marginally correlated with stronger BTAE (*r* = 0.13, *p* < 0.1). Childhood class (*r* = 0.22, *p* < 0.01), and childhood income (*r* = 0.20, *p* < 0.01), were both correlated with stronger BTAE. Education was marginally correlated with BTAE (*r* = 0.13, *p* < 0.1), income was not correlated with BTAE (*r* = 0.10, ns).

Neither self-construal subscale (independence or interdependence) was correlated with any measure of social class, with exception of a negative correlation between SCS Independence and subjective class (5-item measure; *r* = −0.14, *p* < 0.05). SCS Independence was positively correlated with BTAE (*r* = 0.19, *p* < 0.01).

#### Meta-analysis

A meta-analysis of correlations between various measures of SES and BTAE across the 5 studies was conducted (see Table 3). Effect sizes for various measures of SES ranged from 0.05 to −0.25. All SES variables had significant effects on BTAE, with the exception of education.

**Table 3:** **Meta-analysis of Studies 1–5**.

**Measure**	**Mean-weighted *R***	**95% CI**	
SSS	0.25	0.199, 0.299	*p* < 0.001
Class	0.20	0.144, 0.247	*p* < 0.001
Income	0.09	0.037, 0.143	*p* = 0.001
Education	0.04	−0.015, 0.092	*p* = 0.159
Child. Class	0.14	0.091, 0.196	*p* < 0.001
Child. Income	0.15	0.099, 0.204	*p* < 0.001

#### General discussion

In all 5 studies consistent evidence of the BTAE was observed, and in all 5 studies higher SES was linked to a greater tendency to view the self as better than average (see Table 3 for a meta-analysis). Both subjective and objective social class, as well as childhood social class, were linked to stronger BTAE. However, generally speaking the effects were stronger and more consistent for subjective measures of social class than objective measures, and somewhat stronger for current vs. childhood measures of social class. Taken together these findings suggest a robust link between one's SES and the tendency to view the self as better than average. It is not clear from the present studies why some indicators of SES may be more strongly linked to BTAE than others. This issue should be explored further in future research.

However, the 3 studies that manipulated various aspects of status (subjective status, power, and dominance) did not find evidence that temporarily evoked status led to significant differences in BTAE. This result is somewhat surprising as in previous studies manipulations of SES tended to have parallel effects to those of chronic SES. It may be that to some extent the tendency to have an inflated view of one's abilities is more strongly linked to one's environment and chronic experiences, as opposed momentary feelings of rank or status. It may also be that this tendency is somewhat canalized, however if this were the case one would expect correlations between childhood SES and BTAE to be consistently stronger than those between current SES and BTAE. This was not the case. That said, comparing the effect sizes of the manipulations in these studies may still be informative. The power manipulation had an effect roughly twice the size of the manipulation of subjective social status, which hints that power may be a more important component of how people evaluate the self in other domains. The manipulation of dominance (Study 5) had the smallest effect and it was in the opposite of the predicted direction. Future research on status might systematically compare the effects of manipulating different components of status on a number of other social cognitive tendencies that have been linked to status (i.e., empathy, altruism).

The manipulations in Studies 2–4 did not have significant effects on the DV. Further the manipulation in Study 2 did not appear to significantly alter people sense of subjective social status. However, in Studies 3 and 4, the manipulation checks suggested that power and dominance were successfully manipulated. The failure of the manipulation in Study 2 might have been the result of a slight difference in instructions between the manipulation of subjective social status in this study and in those conducted by Kraus. Study 2 in the present research used the instructions provided in the methods section of Kraus et al. (2010). However, the original manipulation included the following additional instructions that were not included in Study 2 in the present MS (Kraus, personal communication):

“Think about an interaction with the person from the top/bottom of the class hierarchy. How is this interaction likely to play out? How are the differences between you likely to shape the way the interaction goes? Please write about this in the space provided.”

Thus, it may be that this failure to include these additional instructions may have reduced the effectiveness of the manipulation in Study 2 (and may account for the failed manipulation check). More generally, stronger manipulations of status (such as assigning different roles in the dictator game), in more controlled settings may still reveal effects parallel to those of chronic SES. All data reported in the present series of studies was gathered using experiments conducted online in which subjects participated in return for relatively minor rewards. It should be noted however that Mturk samples paid similar rewards have been shown to produce high quality psychological data (Buhrmester et al., 2011). Further a recent Many Labs project that sought to replicate 13 classic and contemporary social psychological effects using a mixture of in person and online studies (the latter primarily using Mturk subjects paid small amounts of money) found no systematic variation in experimental effects as a function of whether studies were conducted online or in person (Klein et al., 2014). Nonetheless, it would be worth replicating the present studies in a laboratory setting where experimental control would be greater. In person studies would also provide the opportunity for the use of physiological and neural measures, which may provide further insight into the present findings. Such measures may be more sensitive and also less susceptible to issues including social desirability and lack of insight.

It is also possible that the failure to uncover effects of the experimental manipulation may be due to some unmeasured variables that moderated the effects in such a fashion that they were masked (i.e., Goette et al., 2015). Thus, it would be worthwhile to test effects of potential moderators, such as personality traits, in future research.

It may also be that if we looked at self-evaluations in a fashion that distinguished different components of self-enhancement (i.e., perceiver effects, target effects, and unique self-perception; Kwan et al., 2004) that for certain type(s) of effects chronic and manipulated status might yield more convergent results. This possibility should be explored in future research.

It is also worth noting that Study 5 did not find evidence that SES differences in BTAE were driven by differences self-construal. This is somewhat puzzling given previous studies linking the two (i.e., Na et al., 2010; Grossmann and Varnum, 2011). However, it is worth noting that previous studies documenting this relationship have tended to use implicit or behavioral measures, as opposed to self-report (Na et al., 2010; Grossmann and Varnum, 2011), and that Na et al. (2010) did not observe SES differences on the self-construal scale.

In addition, the samples were confined to Americans. Although some research has found parallel effects of SES in different cultures (Grossmann and Varnum, 2011; Hamamura et al., 2013), it is an empirical question whether the relationship between SES and BTAE is similar in other cultural contexts. Future research should build on the present findings by extending this paradigm to other cultures.

Although the link between SES and BTAE was robust, the mechanism underlying this link remains unclear. Self-construal, at least as measured by self-report, does not appear to be the cause of this relationship. Future research will hopefully shed light on why SES is linked to BTAE. One possibility is that these differences stem from differences in incremental vs. entity beliefs (Kraus and Keltner, 2013). An incremental view may be linked to reduced estimates of one's abilities relative to others, whereas an entity view may have the opposite effect (as this would help defend a high level of self-esteem). This possibility should be explored in future research. It may also be that SES differences in traits like narcissism mediate the effects observed in the present study (Piff, 2014). It would also be interesting to explore lay theories regarding how class is linked to the BTAE, given that previous work (Varnum, 2013), suggests that lay theories regarding class's psychological consequences are not particularly accurate.

Finally, it would interesting to explore whether the gap between actual and perceived abilities might be greater among those higher in SES. One potential alternative explanation is that people who are higher in SES may actually be above average in terms of a number of skills and abilities. However, some previous behavioral findings argue against this possibility. For example, Piff et al. (2010, 2012) has shown in a series of studies that high SES is associated with less ethical behavior and less prosocial behavior, suggesting that the positive correlations observed between SES and self-rated honesty and friendliness in the current study are not likely the result of high SES participants being higher on these dimensions. In fact this work also suggests that the positive correlation observed in the current work between SES and self-reported driving ability is also not likely due to higher SES drivers actually being better drivers. Piff et al. (2012) reports that vehicles belonging to likely high SES drivers (i.e., expensive vehicles) were more likely to commit a traffic infraction. Nonetheless, it is still possible to that higher self-ratings seen among high SES participants in the current studies may reflect some actual ability differences. Future research using behavioral measures as well as self-estimated performance would help to clarify this issue.

### Conflict of interest statement

The author declares that the research was conducted in the absence of any commercial or financial relationships that could be construed as a potential conflict of interest.

## Appendix

### Based on your responses, your personality is likely: leader

You are the type of person whom others look up to. You are often asked for your opinion and people look to you for guidance and advice. You have a natural ability to make decisions and to organize those around you. You thrive in settings where you have responsibility for others. Although you may not always be aware of it, people around you tend to respect you a great deal.

### Based on your responses, your personality is likely: team player

You are the type of person whom others can count on. You are less concerned with getting the credit than you are with getting the job done. You have a natural ability to work well with others and to accomplish the tasks you are given. You thrive in settings where there is structure. Although you may not always be aware of it, people rely on you to do what needs to be done.
